# Liposome nanoparticle conjugation and cell penetrating peptide sequences (CPPs) enhance the cellular delivery of the tau aggregation inhibitor RI‐AG03

**DOI:** 10.1111/jcmm.18477

**Published:** 2024-06-09

**Authors:** Niklas Reich, Edward Parkin, Neil Dawson

**Affiliations:** ^1^ Division of Biomedical and Life Sciences, Faculty of Health and Medicine Lancaster University Lancaster UK

**Keywords:** Alzheimer's disease, cell penetrating peptide, endocytosis, liposomes, nanoparticles, polyarginine, TAT, tau aggregation inhibitor, Tauopathy

## Abstract

Given the pathological role of Tau aggregation in Alzheimer's disease (AD), our laboratory previously developed the novel Tau aggregation inhibitor peptide, RI‐AG03. As Tau aggregates accumulate intracellularly, it is essential that the peptide can traverse the cell membrane. Here we examine the cellular uptake and intracellular trafficking of RI‐AG03, in both a free and liposome‐conjugated form. We also characterize the impact of adding the cell‐penetrating peptide (CPP) sequences, polyarginine (polyR) or transactivator of transcription (TAT), to RI‐AG03. Our data show that liposome conjugation of CPP containing RI‐AG03 peptides, with either the polyR or TAT sequence, increased cellular liposome association three‐fold. Inhibition of macropinocytosis modestly reduced the uptake of unconjugated and RI‐AG03‐polyR‐linked liposomes, while having no effect on RI‐AG03‐TAT‐conjugated liposome uptake. Further supporting macropinocytosis‐mediated internalization, a ‘fair’ co‐localisation of the free and liposome‐conjugated RI‐AG03‐polyR peptide with macropinosomes and lysosomes was observed. Interestingly, we also demonstrate that RI‐AG03‐polyR detaches from liposomes following cellular uptake, thereby largely evading organellar entrapment. Collectively, our data indicate that direct membrane penetration and macropinocytosis are key routes for the internalization of liposomes conjugated with CPP containing RI‐AG03. Our study also demonstrates that peptide‐liposomes are suitable nanocarriers for the cellular delivery of RI‐AG03, furthering their potential use in targeting Tau pathology in AD.

## 1 INTRODUCTION

Alzheimer's disease (AD) pathology is characterized by the extracellular accumulation of amyloid plaques consisting of aggregated amyloid beta (Aβ)‐peptides and the intracellular accumulation of neurofibrillary tangles (NFTs) consisting of aggregated, hyperphosphorylated Tau.^1^ Historically, most therapeutic efforts have focused on the reduction of pathologic Aβ species (reviewed in Pinheiro et al.).^2^ However, the alternative and complementary approach of reducing Tau aggregates has gained considerable traction.^3^ To date, Tau‐targeted therapies have focused on the inhibition of Tau kinases and Tau aggregation, or immunotherapies that enhance clearance of the protein.^3^, ^4^

Under non‐pathological conditions, Tau is a microtubule‐interacting protein localized to neuronal axons.^5^ Through alternative splicing, six Tau isoforms are produced from the *Microtubule‐Associated Protein Tau* (*MAPT*) gene on chromosome 17. Depending on the inclusion of exon 10, Tau isoforms contain either 3 or 4 microtubule‐binding repeat domains (R) along with either zero, one or two N‐terminal inserts (N).^6^, ^7^ Mutations in the *MAPT* gene or the age‐associated build‐up of wild‐type Tau initiates the accumulation, hyperphosphorylation and conformational rearrangement of Tau. This results in microtubular detachment and the sequential aggregation of Tau into toxic soluble oligomers, paired helical filaments (PHFs) and insoluble NFTs within neurons.^6^, ^7^ Tau oligomers and the proteolysed core of PHFs, containing the microtubule repeat domains (R1‐R4), spread between neurons to cross‐seed Tau aggregation.^8^, ^9^, ^10^ This results in the propagation of Tau throughout the central nervous system. Interestingly, the spread of Tau, as assessed by Braak staging in AD,^11^ is more strongly correlated with cognitive decline and the appearance of clinical symptoms than Aβ pathology,^6^, ^7^, ^12^ suggesting that the propagation of Tau is a primary driver of the disease.

Based on the aggregation‐inducing ^16^KLVFF^20^ sequence within Aβ, we previously developed a proteolytically resistant retro‐inverso peptide (RI‐OR2) that reduces Aβ aggregation in an AD cell model and in vivo.^13^, ^14^, ^15^ As conjugating the transactivator of transcription (TAT) cell‐penetrating peptide (CPP) sequence to peptides and nanoparticles improves their blood–brain barrier (BBB) translocation,^16^, ^17^ this CPP was added to RI‐OR2 for in vivo utility.^15^ To further enhance delivery of RI‐OR2‐TAT, the peptide was attached to distearoyl phosphatidylethanolamine (DSPE)‐polyethylene glycol (PEG)‐maleimide (Mal) liposomes to form peptide inhibitor nanoparticles (PINPs).^18^ These PINPs inhibit in vitro Aβ_1‐42_ aggregation more effectively than the unconjugated RI‐OR2 peptide (1:2000 PINP:Aβ vs. 1:5 RI‐OR2‐TAT:Aβ), crossed an in vitro BBB model and improved object recognition memory in Tg2576 (APP_SWE_) mice.^18^

**FIGURE 1:**
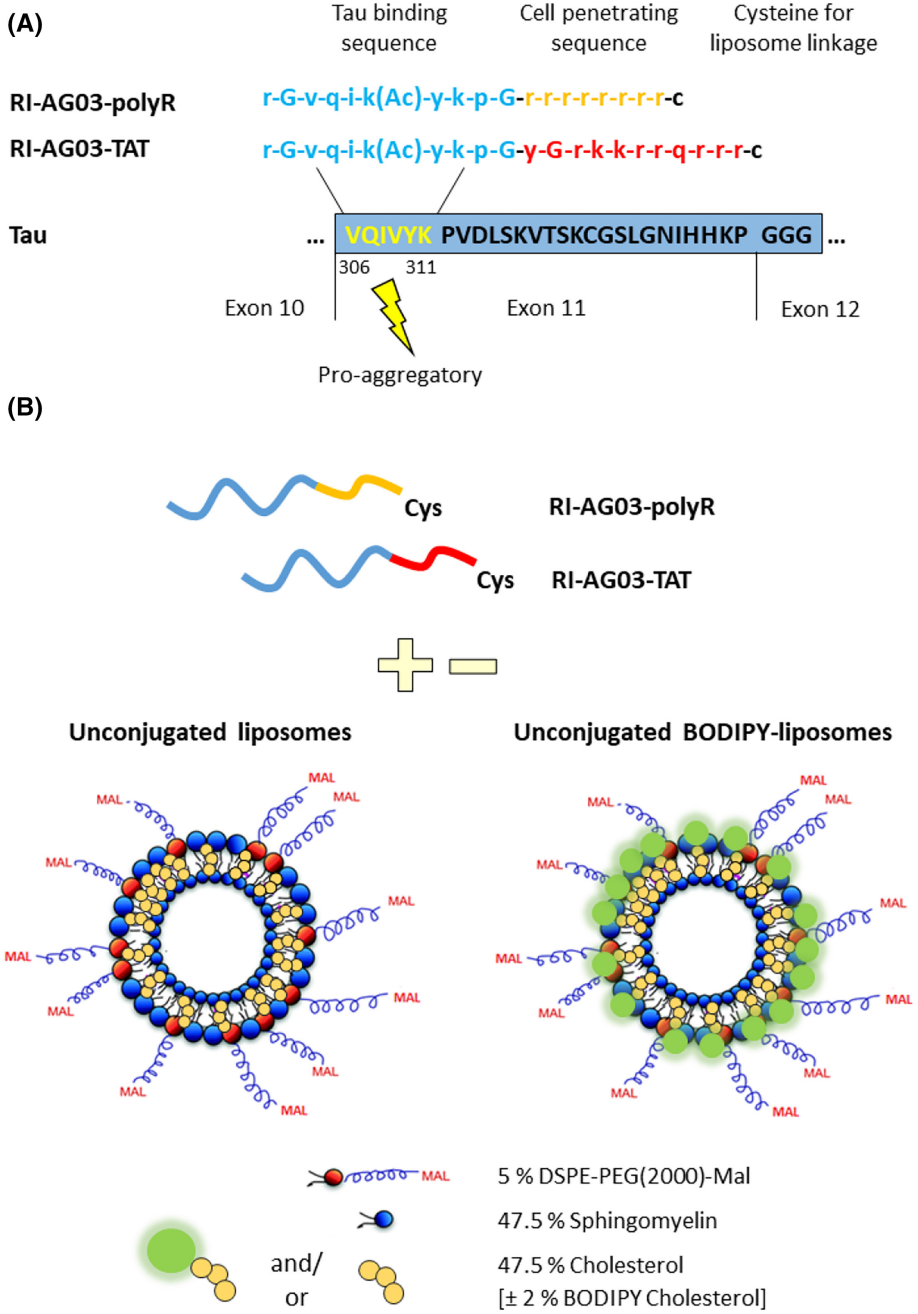
Design of RI‐AG03 derivatives employed in the current study. (A) Sequence of the Tau aggregation inhibitor peptide RI‐AG03. The peptide consists of a Tau binding sequence (blue) that interacts with the pro‐aggregatory ‘VQIVYK’ sequence in microtubule‐binding repeat domain 3 (dark blue box) present in all 6 Tau isoforms. The positively charged cell penetrating sequences polyR (orange) or TAT (red) were attached to the peptide to facilitate cellular uptake. A cysteine was added at the end of the CPP to enable liposome‐linkage via click chemistry. (B) Unconjugated liposomes and BODIPY‐liposomes to which peptides were attached through click chemistry between the cysteine residue of the peptide and protruding maleimide groups (Mal) of the liposomes. For the generation of fluorescent BODIPY‐liposomes, 2% cholesterol was replaced with BODIPY‐cholesterol. Adapted from Chandrasekaran et al.^22^

Given recent interest in Tau as a therapeutic target for AD, we developed a novel peptide that targets Tau aggregation. The ^306^VQIVYK^311^ sequence present in the R3 domain of all six Tau isoforms and the ^275^VQIINK^280^ sequence in the R2 domain present in 4R Tau isoforms drive Tau beta‐sheet formation and aggregation.^19^, ^20^ We previously tested various peptides based on the ^306^VQIVYK^311^ sequence and identified RI‐AG03 (see Figure 1A for sequence) as being particularly effective in attenuating the aggregation of heparin‐seeded recombinant Tau_Δ1‐250_ in vitro, with a 94% reduction in Tau aggregation seen at equimolar drug: Tau concentrations.^21^

Due to the intraneuronal localisation and propagation of Tau,^4^ intracellular delivery of Tau‐targeting therapeutics is essential. Thus, to enhance both BBB penetration and neuronal uptake, CPP sequences can be added to the peptides. Interestingly, octaarginine (polyarginine; polyR) CPP sequence conjugation may also have additional benefits to promoting and BBB penetration,^23^, ^24^, ^25^, ^26^ with arginine‐rich peptides also showing an anti‐aggregatory effect on amyloidogenic peptides (reviewed in Mamsa et al.).^27^ In agreement with this suggestion, we previously found that adding three or nine arginine residues to an earlier version of our peptide (AG02) enhanced inhibition of Tau_Δ1‐250_ aggregation by 20% and 30%, respectively.^21^

Cellular uptake of cargo, including RI‐AG03 and liposomes, may occur in an energy‐independent manner or through ATP‐dependent transport across the cell membrane, also known as endocytosis. Various form of endocytosis exists, such as clathrin‐mediated endocytosis (CME) and clathrin‐independent endocytosis mechanisms, including caveolae‐mediated endocytosis (CavME) and macropinocytosis.^28^, ^29^, ^30^ There are also other less well characterized endocytosis pathways.^28^ CPPs may be taken up by direct membrane translocation, CME, CavME or macropinocytosis.^31^ On the other hand, due to their lipophilic nature, liposomes can directly fuse with and translocate across the cell membrane.^32^, ^33^, ^34^ Conjugation of positively charged CPPs, such as polyR, to liposomes is thought to further improve liposome apposition and fusion with the negatively charged plasma membrane, thus enhancing cellular liposome uptake.^32^ In addition, liposomes may be internalized by endocytosis with multiple characteristics (including cargo type, material composition, size, shape and surface modifications) determining the preferred cellular uptake pathways.^29^, ^31^

While RI‐AG03‐polyR PINPs have previously been shown to prevent the aggregation of recombinant Tau,^21^ their efficacy in cellular models has not been confirmed. In addition, the cellular uptake mechanisms for RI‐AG03‐polyR PINPs have not yet been characterized. Therefore, in this study, we characterize the primary cellular uptake mechanisms and intracellular trafficking of RI‐AG03‐polyR PINPs in SH‐SY5Y cells.

RI‐AG03 was synthesized with either a polyR or TAT sequence to compare the impact of these CPPs on cell internalization (Figure 1). RI‐AG03‐polyR and RI‐AG03‐TAT were attached to liposomes by click chemistry between the cysteine residue of the peptide (added at the end of the CPP sequence) and protruding maleimide groups (Mal) of the liposomes (Figure 1).^35^ To visualize subcellular distribution of the peptide and liposomes, respectively, 6‐FAM and Cy5 peptide derivatives and BODIPY‐cholesterol‐containing liposomes were generated. Endocytosis pathways of unconjugated and peptide‐conjugated BODIPY‐liposomes were evaluated using pharmacological inhibitors, including chlorpromazine (CME), filipin (CavME), cytochalasin D (macropinocytosis and partially CME/CavME) and EIPA (macropinocytosis).^36^, ^37^

Our data show that conjugating RI‐AG03, containing either a polyR or TAT sequence, to liposomes increased cellular liposome association three‐fold. Unconjugated and RI‐AG03 conjugated liposomes were mainly internalized via direct membrane penetration. Cellular uptake of unconjugated and RI‐AG03‐polyR‐linked liposomes was also partially mediated by macropinocytosis. Interestingly, following cell internalization RI‐AG03‐PolyR dissociates from liposomes. Lack of peptide co‐localisation with cell organelles suggests that conjugating RI‐AG03 to liposomes prevents cell organelle entrapment of the peptide. Collectively, these results characterize the cellular uptake mechanisms of our liposome‐conjugated Tau aggregation inhibitor peptides and confirm their intracellular availability as future Tau‐targeted therapeutics.

## 2 MATERIALS AND METHODS

### 2.1 Peptide synthesis

RI‐AG03‐polyR (NH_2_‐r‐G‐v‐q‐i‐k(Ac)‐y‐k‐p‐G‐r‐r‐r‐r‐r‐r‐r‐r‐c), RI‐AG03‐TAT (NH_2_‐r‐G‐v‐q‐i‐k(Ac)‐y‐k‐p‐G‐y‐G‐r‐k–k‐r‐r‐q‐r‐r‐r‐c), 6‐carboxyfluorescein (6‐FAM)‐RI‐AG03‐polyR and 6‐FAM‐RI‐AG03‐TAT were synthesized by Severn Biotech Ltd (Kidderminster, UK). Cyanine‐5 (Cy5)‐RI‐AG03‐polyR was synthesized by Cambridge Peptides Ltd (Cambridge, UK). To allow liposome linkage, all peptides contained an additional cysteine.^35^ The peptides contained D‐amino acids (denoted by lower cases), except for glycine as this amino acid does not possess a D‐enantiomer, to prevent proteolytic cleavage.^14^, ^21^

### 2.2 Liposome preparation

Liposomes were made by dissolving relative molar proportions of the following (all Avanti Polar Lipids Inc., Alabaster, US) in chloroform: 47.5% sphingomyelin (SM; egg‐derived), 47.5% cholesterol (plant‐derived) and 5% maleimide, 1,2‐distearoyl‐sn‐glycero‐3‐phosphoethanolamine‐N‐[maleimide(polyethylene glycol)‐2000] (DSPE‐PEG(2000)‐Mal). For BODIPY‐liposome uptake and localisation fluorescence studies, 2% of the cholesterol was replaced with TopFluor® (BODIPY) cholesterol (Avanti Polar Lipids Inc., Alabaster, US). The lipid mixture was dried under liquid nitrogen and the film resuspended in 1× phosphate‐buffered saline (1× PBS; 137 mmol/L NaCl, 2.7 mmol/L KCl, 20 mmol/L Na_2_HPO_4_ and 1.8 mmol/L KH_2_PO_4_) in a water bath sonicator at 37°C for 15 min. The mixture was then subjected to five freeze–thaw cycles in liquid nitrogen and extruded 11 times using a Mini‐Extruder (Avanti Polar Lipids Inc., Alabaster, US) and Hamilton 1000 μL Syringes (Avanti Polar Lipids Inc., Alabaster, US) through a 0.1 μm Nuclepore™ Polycarbonate Track‐Etched Membrane (Whatman, Maidstone, UK), in accordance with the manufacturer's instructions.

Because DSPE‐PEG(2000)‐Mal is randomly incorporated into liposomes, with the maleimide group facing either inwards or outwards, only half of the DSPE‐PEG(2000)‐Mal present (2.5% liposome lipid content) is available for peptide conjugation. To attach the cysteine residue of RI‐AG03 to the available DSPE‐PEG(2000)‐Mal chains via click chemistry, extruded liposomes were incubated with an excess molar proportion of the peptide (molar concentration of DSPE‐PEG(2000)‐Mal (2.5%) × 1.2) for 2 h at 37°C. The mixture was vortexed once after 1 h and rocked on a plate shaker at room temperature overnight. Unbound peptide was removed by ultracentrifugation for 1 h at 172,000 × g (4°C) and the liposome pellet resuspended in PBS in a water bath sonicator (37°C, three 15 min sonication cycles with vortexing between cycles). To remove any liposome clumps following resuspension, liposomes were centrifuged at (17× *g*) for 4 min, through 0.22 μm Corning® Costar® Spin‐X® centrifuge tube cellulose acetate filters (Corning Inc., Corning, US). Liposome concentrations were then quantified using the LabAssay™ Phospholipid kit (FUJIFILM Wako Shibayagi Corporation, Osaka, Japan).

### 2.3 Cell culture

Human neuroblastoma SH‐SY5Y cells (ATCC®; CRL‐2266; Manassas, USA) were maintained in Dulbecco's Modified Eagle Medium (DMEM)‐F12 (Gibco, Brigg, UK) containing 10% (v/v) heat‐inactivated fetal bovine serum (FBS; Merck Life Science Ltd, Dorset, UK) and 1% (v/v) antibiotic‐antimycotic solution (Merck Life Science Ltd, Dorset, UK) at 37°C and 5% CO_2_.

### 2.4 Flow cytometry

SH‐SY5Y cells were seeded at a density of 350,000 cells per well in 12 well plates and incubated overnight. Cells were then treated with 75 μM unconjugated, RI‐AG03‐polyR‐conjugated or RI‐AG03‐TAT‐conjugated BODIPY‐liposomes for 4 h. To investigate endocytosis pathways, we exposed the SH‐SY5Y cells to inhibitor concentrations previously shown to block endocytosis.^38^, ^39^, ^40^, ^41^ We confirmed that the inhibitor concentrations used were non‐toxic (Figure S1), to ensure that cell death would not affect the measurement of liposome uptake. Cells were treated with vehicle solution (0.5% final dimethyl sulfoxide (DMSO) concentration), 10 μM chlorpromazine hydrochloride, 7.5 μM cytochalasin D, 5 μg/mL filipin III from *Streptomyces filipinensis* or 50 μM 5‐(N‐ethyl‐N‐isopropyl)amiloride (EIPA, all from Merck Life Science Ltd, Dorset, UK) dissolved in DMSO for 30 min prior to co‐incubation with the liposomes for 4 h at 37°C. Cells were then trypsinised, pelleted, washed three times with PBS and resuspended in 1 mL PBS. An equal volume of 4% (w/v) paraformaldehyde (PFA) in PBS was added, for a final concentration of 2% (w/v) PFA, and the cells fixed for 30 min at 4°C in the dark. Following three washes with PBS and resuspension in the same buffer, median cellular fluorescence was quantified by flow cytometry (CytoFLEX; Beckman Coulter, High Wycombe, UK) with a minimum cell count of 10,000 cells per sample. Measurements were performed in triplicate (three wells per group) with three independent repeats using fresh liposome stocks (*n* = 9).

### 2.5 Immunocytochemistry and image analysis

SH‐SY5Y cells were seeded onto poly‐L‐lysine‐coated coverslips at a density of 150,000 cells per well in 24‐well plates and allowed to adhere overnight. Cells were then treated with either 6‐FAM‐RI‐AG03‐polyR/TAT peptide, unconjugated BODIPY‐liposomes, RI‐AG03‐polyR/TAT‐conjugated to BODIPY‐liposomes, 6‐FAM‐RI‐AG03‐polyR/TAT‐conjugated to BODIPY‐liposomes or Cy5‐RI‐AG03‐polyR‐liposomes, and incubated for 2 h or 16 h to investigate cell organelle trafficking. To co‐detect cell organelles, the following live cell stains (Invitrogen, Massachusetts, US) were used according to the manufacturer's instructions; for lysosomes: LysoTracker Deep Red (75 nM), for macropinosomes: pHrodo™ Red Dextran 10,000 Mw (40 μg/mL), for cell membrane: CellLight™ Plasma Membrane‐RFP (25 particles per cell (PPC)), for early endosomes: CellLight™ Early Endosomes‐RFP (25 PPC), for endoplasmic reticulum: CellLight™ ER‐RFP (25 PPC) and for Golgi: CellLight™ Golgi‐RFP (25 PPC). The cells were then washed three times in PBS, fixed in 4% PFA in PBS (4°C for 30 min) and washed three times in PBS. The coverslips were then mounted with ProLong™ Diamond Antifade Mountant containing DAPI (Invitrogen, Massachusetts, US), sealed and stored at 4°C in the dark. At least three images per treatment, from separate coverslips, were taken at 63× magnification using a ZEISS LSM880 confocal microscope (Plan‐APOCHROMAT, 63×/1.40 Oil DIC M27).

To analyse co‐localisation between cell organelles, in the red channel, and the peptides (6‐FAM‐RI‐AG03‐polyR/TAT) or liposomes (BODIPY‐liposomes or 6‐FAM‐RI‐AG03‐polyR/TAT peptides conjugated to liposomes) in the green channel, the Colocalization Threshold plugin of ImageJ was used. The Costes Method was applied to set intensity thresholds. The Pearson's correlation coefficient of co‐localisation (Rcoloc) was subsequently calculated (3–7 images per condition) and qualitatively interpreted (none, poor, fair, moderate, very strong and perfect) in accordance with the definitions outlined by Chan.^42^

### 2.6 Viability assays

SH‐SY5Y cells were seeded in 96 well plates and, at a density of 60%–80% confluency, treated with 75 μM unconjugated liposomes, peptide‐liposomes and endocytosis inhibitors for 4.5 h (*n* = 6; Figure S1). Cell viability was assessed using Cell Counting Kit‐8 (Merck Life Science Ltd, Dorset, UK) according to the manufacturer's instructions.

### 2.7 Statistical analysis

Statistical analysis was performed with JASP (Version 0.18.0, https://jasp‐stats.org/). To compare the cellular uptake of unconjugated and peptide‐conjugated BODIPY‐liposomes in the absence or presence of endocytosis inhibitors, one‐way and two‐way ANOVA followed by Tukey's post‐hoc test were applied. One‐way ANOVA and Tukey's post‐hoc correction were used for the viability assays. Significance was set at *p* < 0.05.

## 3 RESULTS

### 3.1 Conjugating RI‐AG03‐polyR and RI‐AG03‐TAT to liposomes enhances cellular liposome association

**FIGURE 2:**
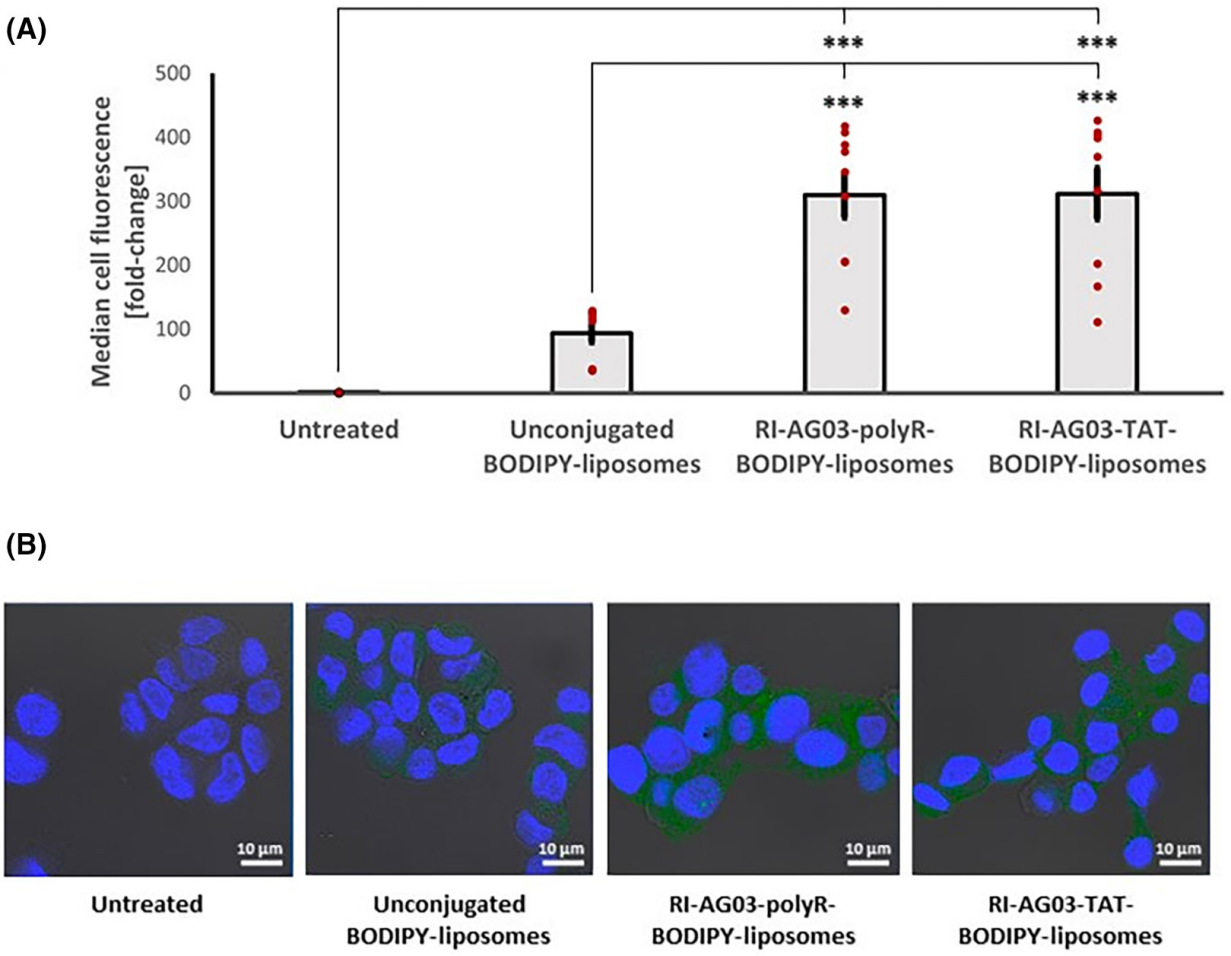
Liposome conjugation with CPP‐containing RI‐AG03 peptides increases cellular liposome association. (A) SH‐SY5Y cells were treated with 75 μM unconjugated, RI‐AG03‐polyR‐conjugated or RI‐AG03‐TAT‐conjugated BODIPY‐liposomes for 4 h and fluorescence quantified via flow cytometry. Data are shown as the Mean ± SEM cell fluorescence (*n* = 9). ***p < 0.001, Tukey's HSD. (B) Representative confocal images of untreated, unconjugated BODIPY‐liposome and peptide‐conjugated BODIPY‐liposome treated SH‐SY5Y cells.

We initially investigated whether unconjugated liposomes or peptide‐conjugated liposomes readily associated with SH‐SY5Y neuroblastoma cells over a 4 h time period. Cells were left untreated or treated with either unconjugated BODIPY‐liposomes, RI‐AG03‐polyR‐conjugated BODIPY‐liposomes or RI‐AG03‐TAT‐conjugated BODIPY‐liposomes. Median cell fluorescence was significantly influenced by treatment type (F_(3,32)_ = 32.42, *p* < 0.001; one‐way ANOVA). While treatment of SH‐SY5Y cells with unconjugated BODIPY‐liposomes led to a 94‐fold increase in median cell fluorescence, this was not significant (Figure 2). By contrast, treatment of cells with RI‐AG03‐polyR‐conjugated and RI‐AG03‐TAT‐conjugated BODIPY‐liposomes resulted in a significant 309‐fold (*p* < 0.001, Tukey's HSD) and 311‐fold (*p* < 0.001, Tukey's HSD) increase, respectively, in median cell fluorescence relative to untreated cells. Conjugating RI‐AG03‐polyR or RI‐AG03‐TAT to BODIPY‐liposomes also significantly increased cellular fluorescence (approximately 3‐fold) relative to unconjugated BODIPY liposomes (*p* < 0.001 in both cases). Therefore, attaching CPP‐containing RI‐AG03 peptides to liposomes significantly increased the cellular association of liposomes.

Confocal microscopy imaging also showed that treatment with unconjugated and peptide‐conjugated BODIPY‐liposomes increased cell‐associated fluorescence (Figure 2B). Cells treated with RI‐AG03‐polyR or RI‐AG03‐TAT‐conjugated BODIPY‐liposomes exhibited an apparent greater fluorescence than that of cells treated with unconjugated BODIPY‐liposomes (Figure 2B), aligned with the quantitative flow cytometry data supporting the enhanced cellular association of the peptide‐conjugated liposomes.

### 3.2 Unconjugated and RI‐AG03‐polyR conjugated liposomes, but not RI‐AG03‐TAT‐conjugated liposomes, are partially internalized by macropinocytosis

**FIGURE 3:**
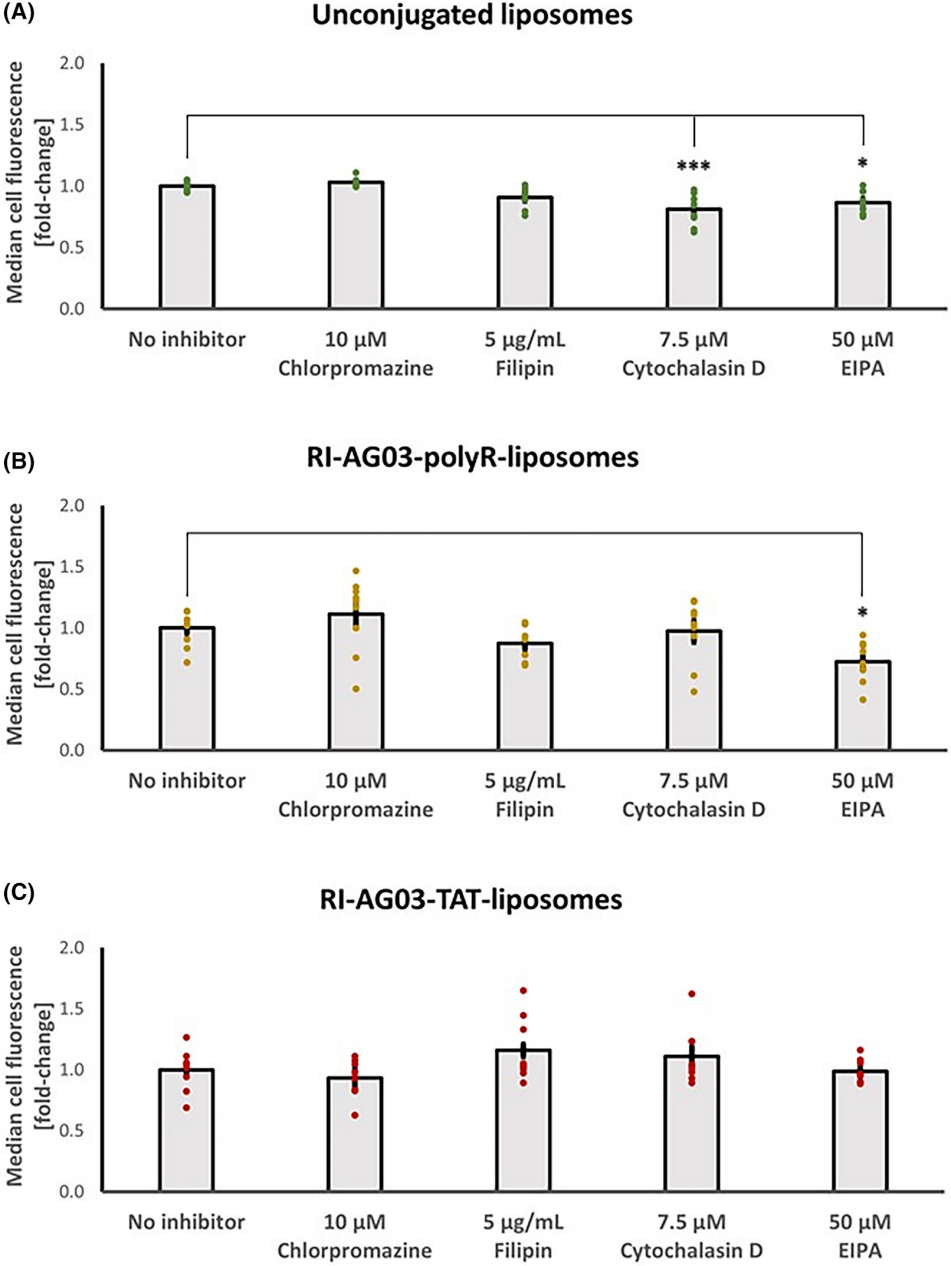
Effects of endocytosis inhibitors on cellular BODIPY fluorescence levels for SH‐SY5Y cells treated with (A) unconjugated, (B) RI‐AG03‐polyR‐conjugated and (C) RI‐AG03‐TAT‐conjugated BODIPY‐liposomes. Data shown as the mean ± SEM median cell fluorescence values normalized to no inhibitor controls (*n* = 9). *p < 0.05 and ***p < 0.001 relative to no‐inhibitor control (post‐hoc Tukey's HSD).

By utilizing the endocytosis inhibitors chlorpromazine, filipin, cytochalasin D and EIPA,^38^, ^39^, ^40^, ^41^ we sought to determine the cellular mechanisms involved in liposome uptake. Endocytosis inhibitor treatment significantly altered BODIPY‐fluorescence levels in SH‐SY5Y cells treated with unconjugated liposomes (F_(4,40)_ = 11.30, *p* < 0.001; one‐way ANOVA; Figure 3A). Both the macropinocytosis inhibitor cytochalasin D (−19%, *p* < 0.001) and the macropinocytosis inhibitor EIPA (−13%, *p* = 0.010) significantly reduced cellular BODIPY fluorescence levels relative to the no‐inhibitor control, when cells were treated with unconjugated BODIPY‐liposomes. By contrast, the CME inhibitor chlorpromazine and CavME inhibitor filipin had no effect on cell BODIPY fluorescence levels when the cells were treated with unconjugated BODIPY‐liposomes. These results suggest that unconjugated BODIPY‐liposomes are partially taken up via macropinocytosis, but that they are not internalized by CME or CavME. However, since the effects of cytochalasin D and EIPA were modest, the majority of unconjugated BODIPY‐liposome uptake by SH‐SY5Y cells appears to occur through energy‐independent membrane fusion and translocation, as previously characterized for liposomes in various other cell types.^29^, ^43^

For RI‐AG03‐polyR‐conjugated BODIPY‐liposomes, only EIPA treatment significantly decreased (−28%) cell BODIPY fluorescence levels relative to no‐inhibitor controls (*p* = 0.027). Chlorpromazine, filipin and cytochalasin D had no effect on cell BODIPY fluorescence levels (Figure 3B). This suggests that RI‐AG03‐polyR‐conjugated BODIPY‐liposome uptake is partly mediated by macropinocytosis, but that it is independent of CME and CavME.

In the case of RI‐AG03‐TAT‐conjugated BODIPY‐liposomes, none of the endocytosis inhibitors altered cellular BODIPY fluorescence levels relative to untreated controls (Figure 3C). Thus, cellular uptake of RI‐AG03‐TAT‐conjugated BODIPY‐liposomes seems to be independent of energy‐dependent macropinocytosis, CME and CavME, and may involve uptake via energy‐independent mechanisms such as direct membrane penetration.

Collectively, these data demonstrate that unconjugated, RI‐AG03‐polyR‐conjugated and RI‐AG03‐TAT‐conjugated BODIPY‐liposome components predominantly associate with cultured SH‐SY5Y cells through direct membrane fusion. However, a small proportion of cellular uptake of unconjugated and RI‐AG03‐polyR‐conjugated liposomes, but not RI‐AG03‐TAT‐conjugated liposomes, involves macropinocytosis.

### 3.3 BODIPY fluorescence from unconjugated and peptide‐conjugated BODIPY‐liposomes localizes to macropinocytosis‐associated cell organelles following cellular uptake

Next we employed immunocytochemistry to investigate how the BODIPY‐labelled cholesterol in our liposomes co‐localized with the cell membrane and organelles following cellular uptake (Table 1). As the cellular distribution of BODIPY‐cholesterol might not reflect that of the peptides bound to the liposomes, we also synthesized fluorescent 6‐carboxyfluorescein (6‐FAM)‐labelled RI‐AG03 peptides to characterize the subcellular distribution of both free and liposome‐conjugated RI‐AG03.

**TABLE 1:** Quantification of peptide, liposome and peptide‐liposome co‐localisation with cell membrane, macropinosome and lysosome markers. Rcoloc = Pearson's correlation coefficient of co‐localisation (*n* = 3–7 images per condition). Qualitative interpretation of Rcoloc based on Chan.^42^

	Cell membrane		Macropinosomes		Lysosomes	
	Rcoloc	Interpretation	Rcoloc	Interpretation	Rcoloc	Interpretation
BODIPY‐liposomes	0.46 ± 0.04	Fair	0.73 ± 0.07	Moderate	0.34 ± 0.01	Fair
RI‐AG03‐polyR‐BODIPY‐liposomes	0.75 ± 0.05	Moderate	0.47 ± 0.12	Fair	0.39 ± 0.06	Fair
RI‐AG03‐TAT‐BODIPY‐liposomes	0.60 ± 0.07	Fair	0.50 ± 0.10	Fair	0.34 ± 0.04	Fair
6‐FAM‐RI‐AG03‐polyR peptide	0.50 ± 0.06	Fair	0.58 ± 0.06	Fair	0.33 ± 0.04	Fair
6‐FAM‐RI‐AG03‐TAT peptide	0.30 ± 0.11	Fair	0.54 ± 0.17	Fair	0.14 ± 0.07	Poor
6‐FAM‐RI‐AG03‐polyR‐liposomes	0.54 ± 0.07	Fair	0.33 ± 0.21	Fair	0.08 ± 0.25	None
6‐FAM‐RI‐AG03‐TAT‐liposomes	0.39 ± 0.10	Fair	−0.02 ± 0.05	Poor	−0.03 ± 0.04	None

**FIGURE 4:**
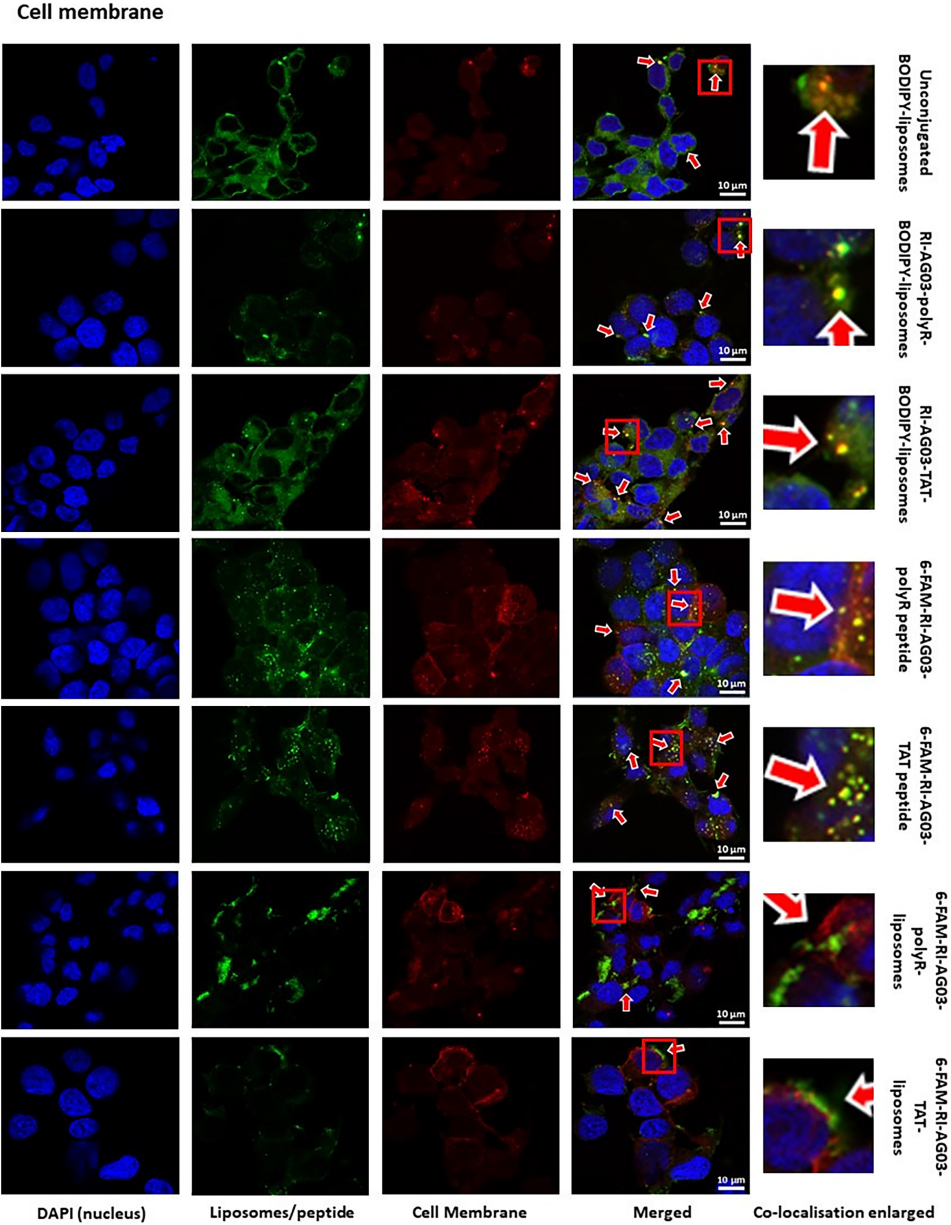
Co‐localisation of BODIPY (liposome) and 6‐FAM (peptide) fluorescence from unconjugated and RI‐AG03‐conjugated liposomes with the cell membrane. SH‐SY5Y cells were co‐treated with unconjugated or RI‐AG03‐polyR/TAT‐conjugated BODIPY‐liposomes (green fluorescence liposomes) or unconjugated 6‐FAM‐RI‐AG03‐polyR/TAT peptide and 6‐FAM‐RI‐AG03/TAT‐conjugated liposomes (green fluorescent peptide) and CellLight™ Plasma Membrane‐RFP (red) for 16 h. Arrows highlight observed co‐localisation.

BODIPY fluorescence from unconjugated and RI‐AG03‐polyR/TAT‐conjugated BODIPY‐liposomes showed fair to moderate co‐localisation with the plasma membrane marker CellLight™ Plasma Membrane‐RFP after 16 h of incubation (Table 1; Figure 4). Similar to BODIPY, unconjugated and liposome‐conjugated 6‐FAM‐RI‐AG03‐polyR and 6‐FAM‐RI‐AG03‐TAT also displayed fair co‐localisation with the cell membrane at 16 h of incubation (Table 1; Figure 4). This suggests that, at the 16 h incubation timepoint, a proportion of the liposome vehicle and RI‐AG03 peptides are integrated into, or in transit through, the cell membrane.

**FIGURE 5:**
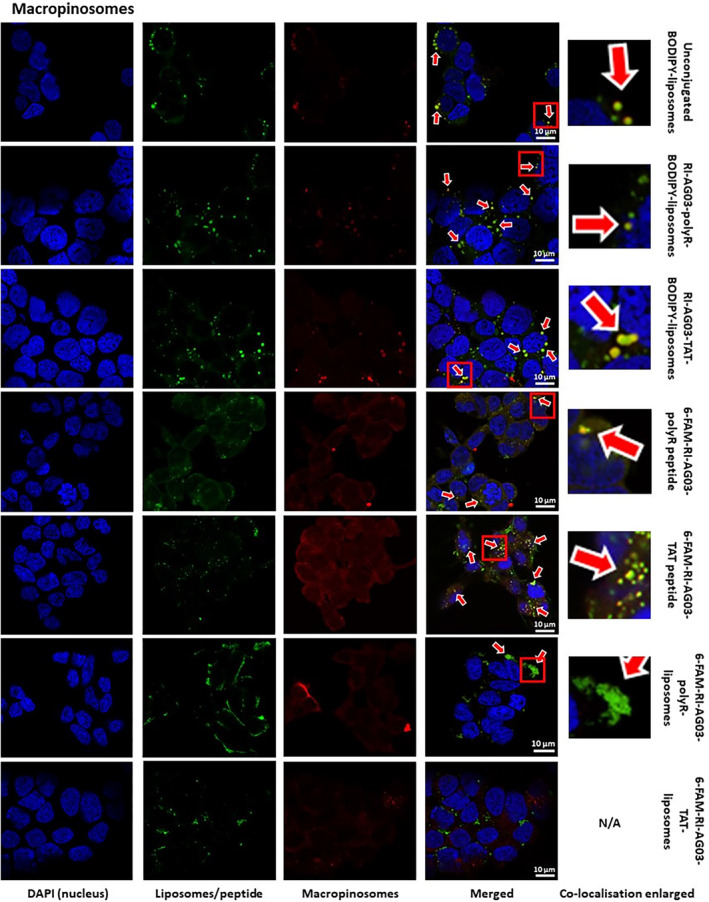
Co‐localisation of BODIPY fluorescence from unconjugated and peptide‐conjugated BODIPY‐liposomes, and 6‐FAM fluorescence from free or liposome‐conjugated RI‐AG03‐polyR/TAT peptides, with macropinosomes in SH‐SY5Y cells. Cells were co‐incubated with BODIPY‐liposomes (green), 6‐FAM labelled peptides (green) and 6‐FAM peptide conjugated liposomes (green) and pHrodo™ Red Dextran (macropinosomes, red) for 16 h. Arrows indicate observed co‐localisation.

Regarding cell organelles, image analysis revealed that BODIPY from liposomes showed moderate (unconjugated BODIPY‐liposomes) or fair (RI‐AG03‐polyR‐BODIPY‐liposomes and RI‐AG03‐TAT‐BODIPY‐liposomes) co‐localisation with macropinosomes (pHrodo™ Red Dextran, Table 1; Figure 5). The modest co‐localisation of BODIPY with macropinosomes parallels our earlier results, which showed internalization of these BODIPY‐liposomes by macropinocytosis (Figure 3). While BODIPY from RI‐AG03‐TAT‐conjugated BODIPY‐liposomes had fair co‐localisation with macropinosomes (pHrodo™ Red Dextran), despite our data supporting endocytosis‐independent internalization (Figure 3C), this co‐localisation appeared to be weaker than that of unconjugated BODIPY‐liposomes (Table 1).

Similar to the fair co‐localisation of BODIPY from peptide‐conjugated liposomes with macropinosomes, unconjugated 6‐FAM‐RI‐AG03‐polyR and 6‐FAM‐RI‐AG03‐TAT peptides also showed fair macropinosome co‐localisation (pHrodo™ Red Dextran, Table 1; Figure 5). This suggests that both the liposome vehicle and free RI‐AG03‐polyR peptides partially traffic into macropinosomes when internalized by SH‐SY5Y cells. However, while 6‐FAM‐RI‐AG03‐PolyR also showed fair co‐localisation to macropinosomes when conjugated to liposomes, 6‐FAM‐RI‐AG03‐TAT conjugated to liposomes showed poor co‐localisation with macropinosomes (Table 1; Figure 5). This contrasts with the fair co‐localisation of BODIPY from 6‐FAM‐RI‐AG03‐TAT conjugated liposomes with macropinosomes. Thus, the RI‐AG03‐TAT peptide may undergo a different subcellular distribution to the liposome vehicle that it is conjugated to.

**FIGURE 6:**
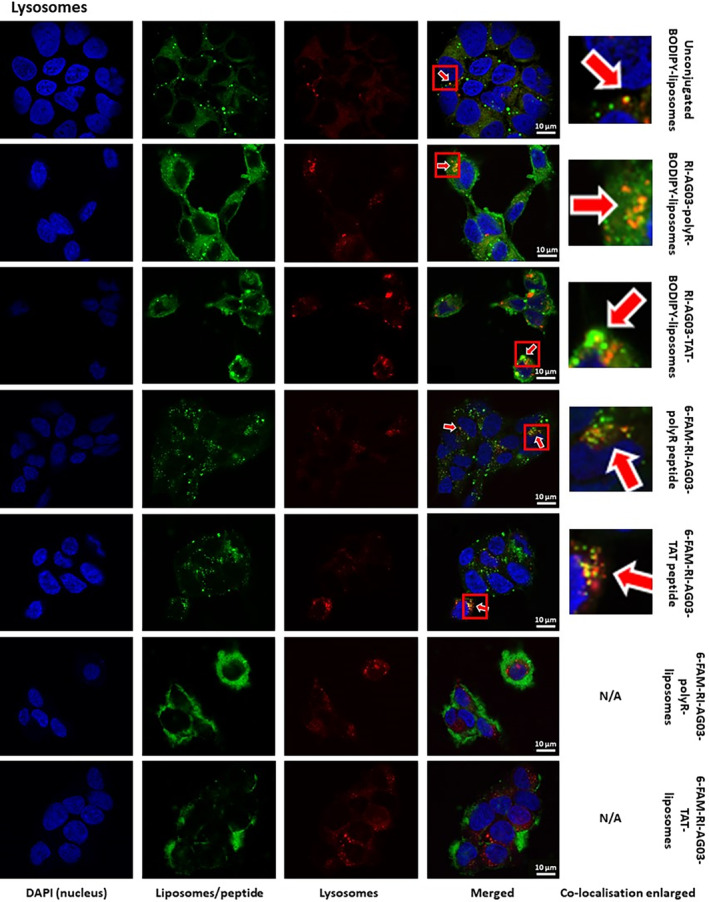
Co‐localisation of BODIPY fluorescence from unconjugated and peptide‐conjugated BODIPY‐liposomes, and free and liposome‐conjugated 6‐FAM‐RI‐AG03‐polyR/TAT peptides (fluorescent 6‐FAM‐peptide), with lysosomes in SH‐SY5Y cells. Cells were co‐incubated with BODIPY‐liposomes (green), 6‐FAM labelled peptides (green) and 6‐FAM peptide conjugated to liposomes (green) and LysoTracker Deep Red (liposomes, red) for 16 h. Arrows indicate observed co‐localisation.

BODIPY from all three liposome types displayed fair co‐localisation with lysosomes (LysoTracker Deep Red, Table 1; Figure 6). The free forms of both 6‐FAM‐RI‐AG03‐polyR and 6‐FAM‐RI‐AG03‐TAT showed fair and poor co‐localisation with lysosomes, respectively. Interestingly, in contrast to BODIPY fluorescence from peptide‐conjugated BODIPY‐liposomes, there was no co‐localisation of the 6‐FAM‐labelled peptides with lysosomes when conjugated to these liposomes (Table 1; Figure 6). This suggests that the liposome vehicle, but not the conjugated RI‐AG03‐polyR/TAT peptide, undergoes processing in lysosomes. The data also suggest that peptide conjugation to the liposomes reduces lysosomal trafficking in comparison to when the free peptides are applied.

To further explore the localisation of the liposome‐conjugated 6‐FAM peptides, co‐localisation with fluorescent probes targeting other cell organelles was also characterized. There was no convincing co‐localisation with early endosomes, the endoplasmic reticulum or the Golgi (Supplemental Material, Table S1; Figures S2–S4). Collectively, these data suggest that the liposome vehicle and the initially conjugated peptides have distinct intracellular trafficking patterns during and after their internalization by SH‐SY5Y cells.

### 3.4 RI‐AG03 dissociates from liposomes following cellular membrane fusion, thus leading to disparate localisation of the liposome carrier and the initially conjugated peptide

The disparate co‐localisation of 6‐FAM‐peptide conjugated to liposomes and BODIPY from peptide‐conjugated liposomes with macropinosomes and liposomes led us to investigate whether the peptide detaches from its liposome vehicle before moving to alternative cellular destinations. Therefore, we used a Cy5‐labelled RI‐AG03‐polyR peptide (red) linked to BODIPY‐liposomes (green) to simultaneously monitor the subcellular distribution of the peptide and the liposomal carrier in the same cells, following 2 and 16 h incubations.

**FIGURE 7:**
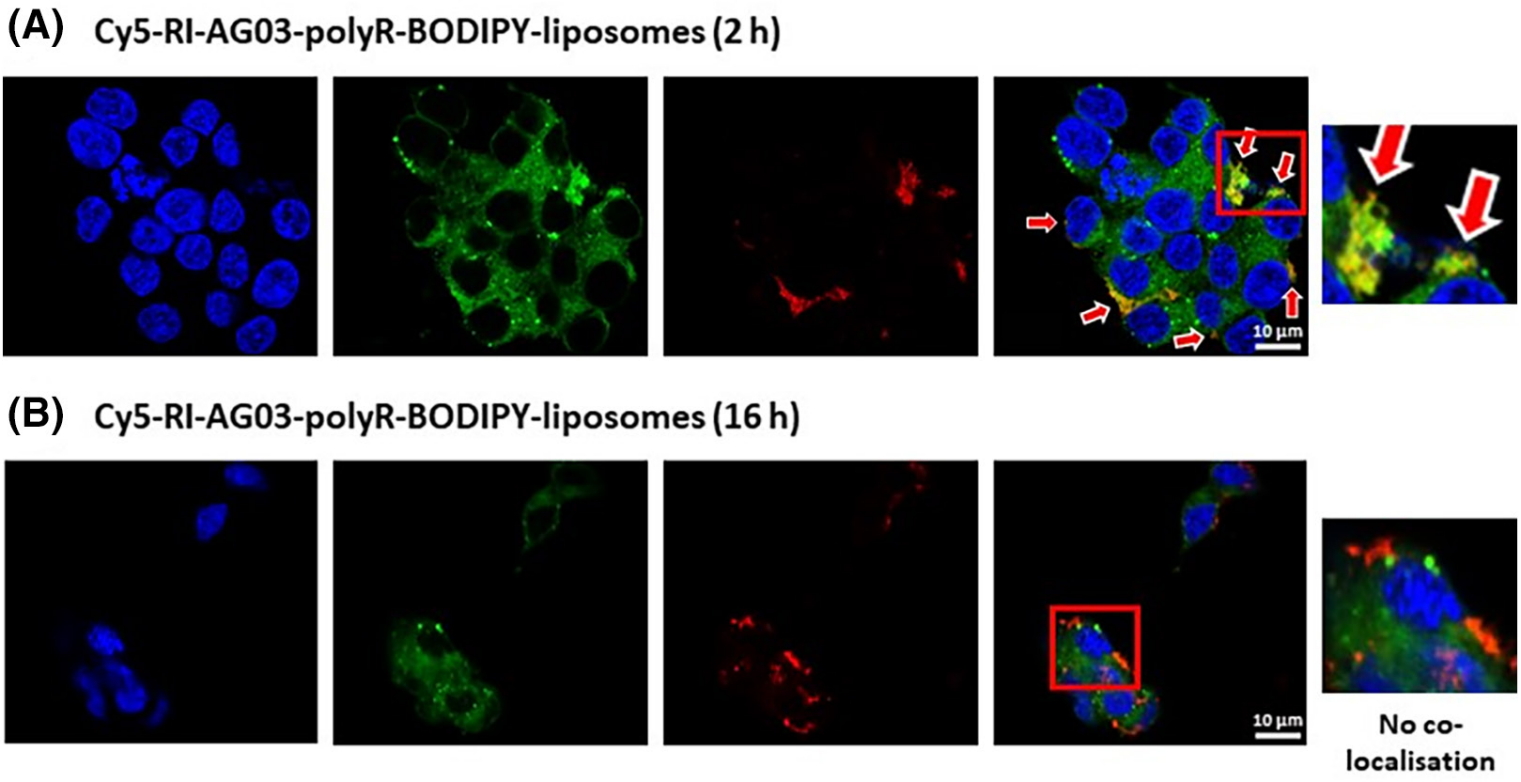
Dissociation of Cy5‐RI‐AG03‐polyR from its liposome carrier following fusion with cells. SH‐SY5Y cells were treated with Cy5‐RI‐AG03‐polyR‐BODIPY‐liposomes for 2 h or 16 h. Red indicates the Cy5‐RI‐AG03‐polyR peptide and green indicates BODIPY fluorescence. Arrows indicate observed co‐localisation.

After a 2 h treatment with Cy5‐RI‐AG03‐polyR‐BODIPY‐liposomes, co‐localisation of the peptide and liposome carrier was fair (Rcoloc = 0.5962, Figure 7A). However, after 16 h there was no co‐localisation between Cy5‐RI‐AG03‐polyR and BODIPY (Rcoloc = −0.0186, Figure 7B). This further suggest that, following membrane fusion and cellular uptake, the peptide dissociates from the liposome carrier.

## 4 DISCUSSION

This study demonstrates that RI‐AG03‐polyR and RI‐AG03‐TAT conjugation to liposomes enhances liposome association with SH‐SY5Y cells (Figure 2). The high cholesterol content of our liposomes (47.5%) likely facilitates their cellular uptake, as incorporating cholesterol into liposomes increases uptake by SH‐SY5Y cells, BBB‐associated brain microvascular endothelial cells and glia‐like Schwann cells. By contrast, cholesterol incorporation did not increase liposome uptake by skeletal muscle‐like NIH‐3 T3 fibroblasts.^44^ This suggests that the lipid composition of our liposomes might favour BBB translocation and fusion with both Tau‐containing neurons and oligodendrocytes (the CNS counterpart of peripheral Schwann cells)^45^, ^46^ over uptake by skeletal muscle cells.

The surface charge of the liposomes, which can be modified by CPP‐containing peptide conjugation, also impacts cellular uptake.^29^ Positively charged (cationic) nanoparticles are electrostatically attracted to negatively charged (anionic) bilipid membranes, such as the cell membrane.^47^ We previously characterized the zeta potential of highly similar liposome formulations, and our studies indicate that the unconjugated liposomes used in this study (47.5% cholesterol, 47.5% SM and 5% DSPE‐PEG(2000)‐Mal) exhibit a negative surface charge.^48^ However, polyR and TAT are positively charged CPPs (net charge +8),^49^ with RI‐AG03‐polyR and RI‐AG03‐TAT proteins predicted to exhibit an overall positive charge of +10.9 (PepCalc, Innovagen AB). Thus, it is likely that conjugation of these peptides to our liposomes imparts a positive charge to the surface. Indeed, PolyR peptide conjugation to liposomes has previously been shown to impart positive surface charge to liposomes with an initially negative zeta potential,^50^ thus improving fusion with the cationic cell membrane.^32^ Future work characterizing the zeta potential of our different liposomes would be of interest to confirm that these are indeed positively charged. As such, RI‐AG03‐polyR and RI‐AG03‐TAT conjugation to our liposomes could electrostatically enhance the integration of liposomes with the cell membrane of SH‐SY5Y cells. This charge effect might contribute to the 3‐fold enhanced uptake of RI‐AG03‐polyR‐ and RI‐AG03‐TAT‐conjugated BODIPY‐liposomes relative to unconjugated BODIPY‐liposomes (Figure 2). In agreement with this, the surface conjugation of polyR to PEG(2000)‐containing liposomes resulted in greater transfection of H4II‐E cells as compared to non‐polyR‐coated liposomes.^51^ In addition, coating polyethylenimine/PEG‐liposomes with TAT peptides also increases the transfection efficiency of SH‐SY5Y cells.^52^ However, to further clarify the contribution of the electrostatic protein properties and the specific effects of the different CPP sequences in the RI‐AG03 peptides to the enhanced liposome uptake seen further systematic investigation is required.

We found that SH‐SHY5Y cells partially internalized unconjugated liposomes and RI‐AG03‐polyR‐conjugated liposomes through macropinocytosis (Figure 3). Macropinocytosis involves the formation of cell membrane protrusions (lamellipodia) that engulf large volumes of extracellular fluid. This nonselective mechanism enables cellular uptake of molecules that are too large for other endocytosis pathways. Internalized macropinosomes range from 0.5–10 μm in diameter and are, in part, delivered to lysosomes.^29^, ^53^ The moderate and fair co‐localisation of BODIPY fluorescence from unconjugated and RI‐AG03‐polyR‐conjugated liposome, respectively, with macropinosomes and lysosomes supports a role for this cellular uptake and processing mechanism in SH‐SY5Y cells (Table 1; Figure 5). While the internalization of BODIPY from RI‐AG03‐TAT‐BODIPY‐liposomes appeared to be largely independent of macropinocytosis in SH‐SY5Y cells (Figure 3), there was fair co‐localisation of BODIPY from these liposomes with macropinosomes and lysosomes (Table 1; Figure 5). As expected, unconjugated BODIPY‐liposomes, whose uptake was partially dependent on macropinocytosis, showed greater (moderate) co‐localisation with macropinosomes than RI‐AG03‐TAT‐BODIPY‐conjugated liposomes. Therefore, it is possible that random membrane fusion events, as is typical for liposomes,^29^, ^43^ may contribute to the colocalization of BODIPY from RI‐AG03‐TAT‐conjugated BODIPY‐liposomes with macropinocytosis‐associated (macropinosomes and lysosomes) cell organelles. Our data suggest that RI‐AG03‐TAT conjugated liposome uptake is independent of the ATP‐dependent endocytosis mechanisms characterized in these studies. Given that neurons are bioenergetically impaired in AD, as indicated by impaired glucose metabolism and insulin resistance,^54^, ^55^ the cellular uptake of tau targeting peptides by energy‐independent mechanisms, such as direct cell penetration, may be advantageous.

Treatment of SH‐SY5Y cells with RI‐AG03‐polyR‐conjugated BODIPY‐liposomes and the CME inhibitor chlorpromazine appeared to slightly increase median cell‐associated fluorescence (11.12%, Figure 3B), suggesting potentially enhanced cellular uptake of these liposome. However, this effect was found to be non‐significant (*p* = 0.132), suggesting that CME inhibition does not influence the cellular uptake of these liposomes. A similar effect was reported by Lee et al., who found a non‐significant increase in liposome‐associated cellular fluorescence when Schwann cells were treated with the CME inhibitor promazine.^44^ Overall, the data suggest that cellular liposome uptake is not influenced by CME inhibition, a contention also supported by our observations in unconjugated and RI‐AG03‐TAT conjugated liposomes (Figure 3A,C).

We found that the different CPPs present in the RI‐AG03 peptide affected the cellular uptake mechanisms involved, consistent with published data. Nona‐arginine peptides induce cell membrane multi‐lamellarity and increase energy‐independent cellular uptake.^56^ The surface conjugation of polyR to liposomes also enhances the apposition and fusion of liposomes with lipid bilayers, promoting uptake.^32^, ^33^ In addition, high concentrations (40 μM) of nona‐arginine and TAT promote uptake through direct membrane penetration and macropinocytosis rather than through CME.^57^ This suggests that coating liposomes with polyR‐ or TAT‐containing peptides acts via various cell uptake mechanisms to enhance liposomal cell association and uptake, consistent with the 3‐fold increase in liposome association found in our study for liposomes conjugated with the CPP‐containing peptides (Figure 2). For the RI‐AG03‐TAT peptide, this appeared to occur in an endocytosis‐independent manner (Figure 3C). By contrast, the increased uptake of RI‐AG03‐polyR‐conjugated liposomes was partially mediated by increased macropinocytosis (Figure 3). However, this represented only a small proportion of cellular uptake. Thus, our data show that the presence of CPP‐containing peptides can enhance cellular liposome uptake by both energy‐independent and endocytosis‐mediated uptake, dependent upon the CPP present.

One important limitation to our study is that we are unable to investigate several less well characterized endocytosis mechanisms, such as CLIC/GEEC‐driven endocytosis, flotillin‐mediated endocytosis and circular dorsal ruffles,^28^ as specific inhibitors for these endocytic pathways are currently lacking.^36^, ^37^ Thus, we cannot rule out liposome uptake by these alternative endocytosis mechanisms in SH‐SY5Y cells.

A major challenge for drug‐conjugated nanocarriers is to avoid entrapment in cell degradative compartments, such as endosomes and lysosomes.^33^ It has been proposed that the linkage of polyR, TAT and other CPPs to liposomes facilitates endolysosome membrane fusion, leading to the ejection of liposome encapsulated cargo into the cytoplasm.^32^ However, in some circumstances the inclusion of CPPs may in fact facilitate organelle entrapment. For example, Ruan et al. demonstrated that TAT‐conjugated quantum dots internalized by macropinocytosis become trapped in the inner macropinosome membrane.^58^ Thus, linking CPPs to nanocarriers may not necessarily improve cell organelle escape, and using additional organelle escape strategies might be necessary.^32^ In this study we found that BODIPY from unconjugated, RI‐AG03‐polyR conjugated and RI‐AG03‐TAT conjugated BODIPY‐liposomes had fair to moderate co‐localisation with the cell membrane, macropinosomes and lysosomes in SH‐SY5Y cells (Table 1). 6‐FAM‐labelled RI‐AG03‐polyR and RI‐AG03‐TAT conjugated to non‐fluorescent liposomes also displayed a comparable (fair) level of co‐localisation with the cell membrane (Table 1; Figure 4). However, these peptides showed no/poor co‐localisation with lysosomes, early endosomes, the ER and Golgi when conjugated to liposomes. In addition, we found that 6‐FAM‐RI‐AG03‐polyR conjugated to BODIPY‐labelled liposomes detached from the liposomes after fusing with the SH‐SY5Y cell membrane (Figure 7). Overall, these data suggest that, when the peptide‐liposomes are internalized, the conjugated peptide dissociates from the liposome vehicle and, at least partially, escapes entrapment in degrative cell organelles, potentially facilitating the dispersal of the peptide throughout the cytoplasm. Given that unconjugated 6‐FAM‐RI‐AG03‐polyR/TAT peptides exhibited a higher co‐localisation with macropinosomes, lysosomes and early endosomes than when the peptides were conjugated to liposomes (Table 1), this suggests that peptide‐conjugation to liposomes may alter peptide trafficking in favour of enhanced cytoplasmic delivery. However, further studies are necessary to investigate the cytoplasmic location of the peptides and their potential interaction with cytoplasmic Tau.

## 5 CONCLUSION

Conjugating CPP containing RI‐AG03 to liposomes, independent of the CPP sequence (TAT or polyR) included in the peptide, enhances SH‐SY5Y cellular association. While RI‐AG03‐polyR‐conjugated liposomes are partially internalized by macropinocytosis, cellular uptake of RI‐AG03‐TAT‐conjugated liposomes is not significantly mediated by the ATP‐dependent endocytosis mechanisms characterized in this study. Thus, RI‐AG03‐TAT is potentially the preferential choice for cellular delivery, given the bioenergetic impairment of neurons in AD and Tauopathies. We also found that RI‐AG03‐PolyR detaches from its liposome carrier following cellular uptake, and that its conjugation to liposomes avoids peptide entrapment in degrading cell organelles. This suggests that liposome conjugation also enhances the cellular availability of RI‐AG03, which may increase its utility in preventing cellular tau aggregation. Future work is needed to confirm this hypothesis. Overall, our data suggest that RI‐AG03 conjugation to liposomes, to form PINPs, may be useful in enhancing the cellular availability of the peptide, and thus may be beneficial in the use of the peptide for the treatment of Tau pathology in neurodegenerative diseases.

## AUTHOR CONTRIBUTIONS

**Niklas Reich:** Formal analysis (equal); investigation (equal); writing – original draft (equal). **Edward Parkin:** Project administration (equal); supervision (equal); writing – review and editing (equal). **Neil Dawson:** Conceptualization (equal); data curation (equal); funding acquisition (equal); project administration (equal); supervision (equal); writing – review and editing (equal).

## FUNDING INFORMATION

The research was supported by Lancaster University's Defying Dementia charity (https://www.lancaster.ac.uk/giving/defying‐dementia/).

## CONFLICT OF INTEREST STATEMENT

Niklas Reich, Edward Parkin and Neil Dawson have no conflict of interest.

## Supporting information


Data S1.

## Data Availability

The data that support the findings of this study are available from the corresponding author upon reasonable request.
