# Supplementary material for: Liposome nanoparticle conjugation and cell penetrating peptide sequences (CPPs) enhance the cellular delivery of the tau aggregation inhibitor RI‐AG03
Source: J Cell Mol Med. 2024 Jun 9;28(11):e18477. doi: 10.1111/jcmm.18477 (PMC11163028; doi:10.1111/jcmm.18477)
Supplement: Supplementary file 1 — Data S1. [file JCMM-28-e18477-s001.docx]

**Supplementary Material**

***Table S1: Quantification of peptide, liposome and peptide-liposome co-localisation with early endosomes, the endoplasmic reticulum and Golgi.*** *Rcoloc = Pearson’s correlation coefficient (n = 3 - 7 images were analysed per condition). Interpretation of co-localisation was according to Chan (2003).*

|  | Early endosomes | | Endoplasmic reticulum | | Golgi | |
| --- | --- | --- | --- | --- | --- | --- |
|  | **Rcoloc** | **Interpretation** | **Rcoloc** | **Interpretation** | **Rcoloc** | **Interpretation** |
| BODIPY-liposomes | 0.47 ± 0.07 | Fair | 0.27 ± 0.06 | Poor | 0.37 ± 0.09 | Fair |
| RI-AG03-polyR-BODIPY-liposomes | 0.29 ± 0.04 | Poor | 0.26 ± 0.06 | Poor | 0.11 ± 0.12 | Poor |
| RI-AG03-TAT-BODIPY-liposomes | 0.31 ± 0.06 | Fair | 0.25 ± 0.09 | Poor | 0.24 ± 0.09 | Poor |
| 6-FAM-RI-AG03-polyR peptide | 0.43 ± 0.04 | Fair | 0.04 ± 0.06 | None | -0.03 ± 0.06 | None |
| 6-FAM-RI-AG03-TAT peptide | 0.11 ± 0.05 | Poor | 0.18 ± 0.11 | Poor | -0.14 ± 0.05 | None |
| 6-FAM-RI-AG03-polyR-liposomes | -0.14 ± 0.07 | None | -0.10 ± 0.03 | None | -0.09 ± 0.06 | None |
| 6-FAM-RI-AG03-TAT-liposomes | 0.09 ± 0.08 | None | 0.17 ± 0.10 | Poor | -0.03 ± 0.10 | None |


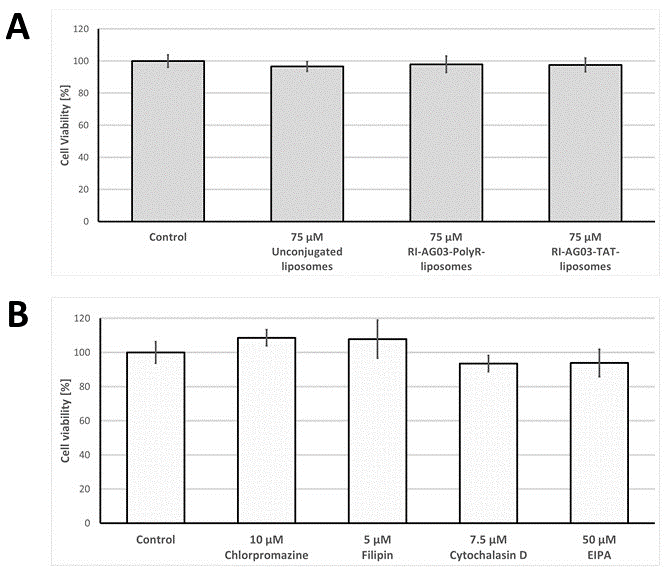


***Figure S1: Toxicity analysis of liposomes, peptide-liposomes and endocytosis inhibitors.*** *SH-SY5Y cells were treated with the indicated final concentrations of (****A****) unconjugated, RI-AG03-polyR-conjugated or RI-AG03-TAT-conjugated liposomes and (****B****) endocytosis inhibitors for 4.5 h. Data show average cell viability in comparison to vehicle-treated controls (n = 6; Mean ± SEM), as assessed by the conversion of water-soluble tetrazolium salt-8 (WST-8). There were no significant differences from vehicle-treated controls (post-hoc Tukey’s HSD).*


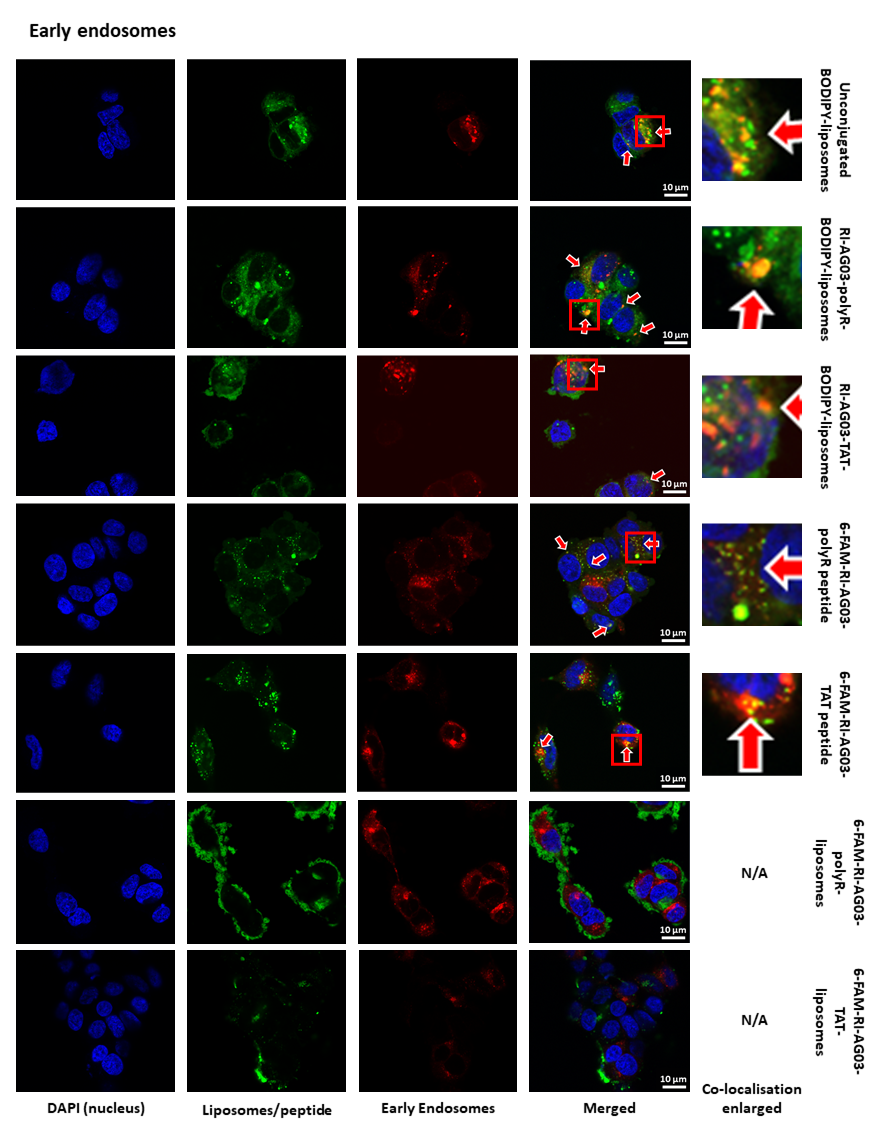


***Figure S2: Co-visualisation of RI-AG03, unconjugated liposomes and RI-AG03-conjugated liposomes with early endosomes.*** *SH-SY5Y cells were treated with the indicated constructs (either with free or conjugated fluorescent 6-FAM peptides or BODIPY cholesterol in liposomes; in green) and Rab5a-RFP (CellLight™ Early Endosomes-RFP; in red) for 16 h. Nuclei were stained with DAPI (blue). Arrows indicate observed co-localisation.*


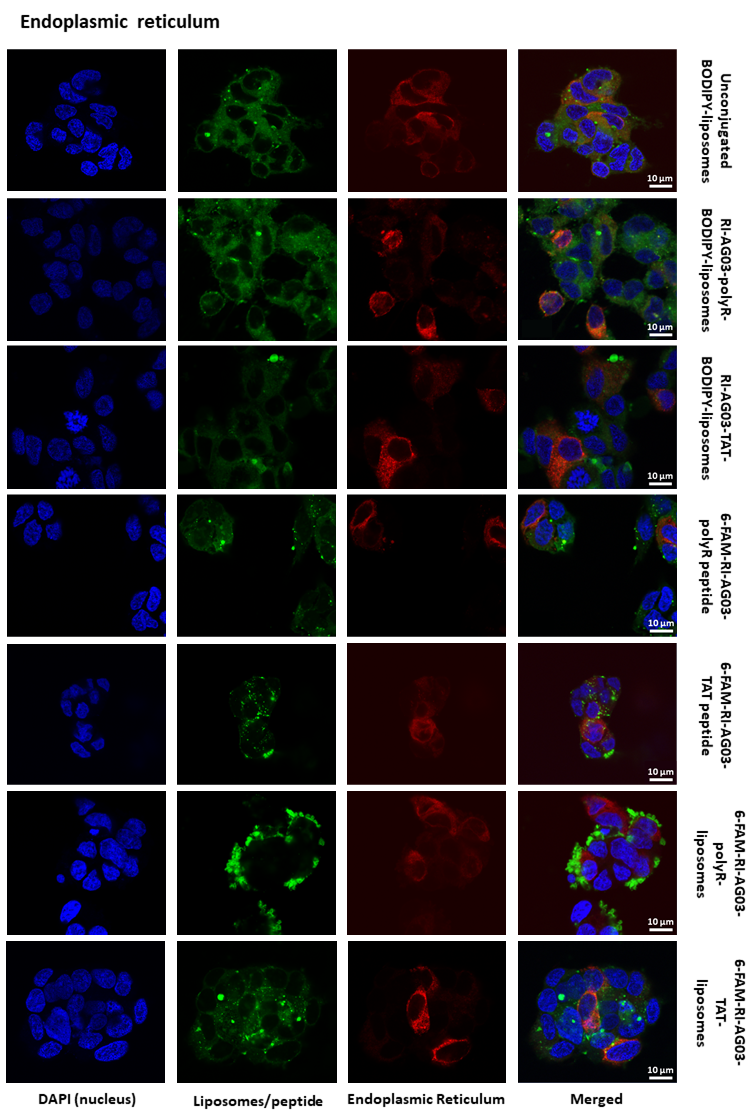


***Figure S3: Co-localisation of RI-AG03, unconjugated liposomes and RI-AG03-conjugated liposomes with the endoplasmic reticulum.*** *SH-SY5Y cells were treated with the indicated constructs (either with free or conjugated fluorescent 6-FAM peptides or BODIPY cholesterol in liposomes; in green) and CellLight™ ER-RFP (in red) for 16 h. Nuclei were stained with DAPI (blue).*


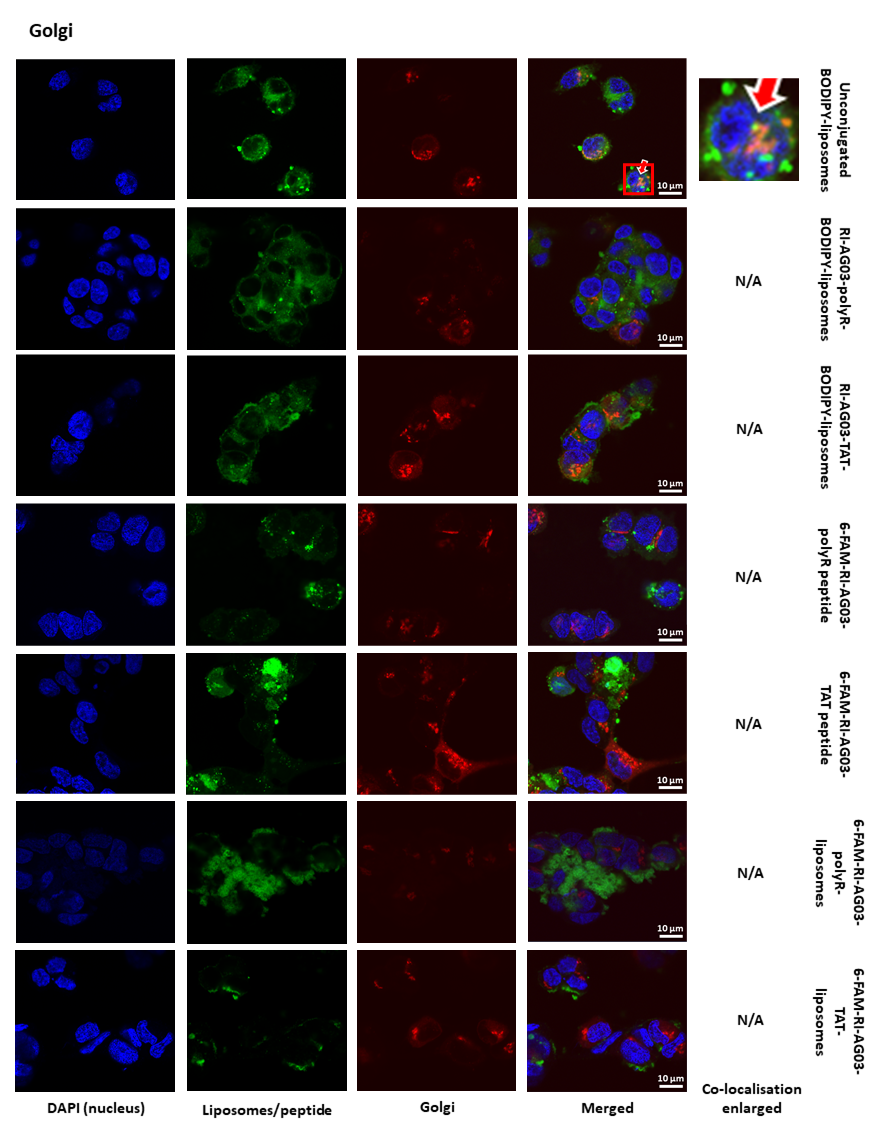


***Figure S4: Co-localisation of RI-AG03, unconjugated liposomes and RI-AG03-conjugated liposomes with the Golgi.*** *SH-SY5Y cells were treated with the indicated constructs (either with free or conjugated fluorescent 6-FAM peptides or BODIPY cholesterol in liposomes; in green) and N-acetylgalactosaminyltransferase-RFP (CellLight™ Golgi-RFP, in red) for 16 h. Nuclei were stained with DAPI (blue). Arrows show observed co-localisation.*
